# The molecular epidemiology and antimicrobial resistance of *Staphylococcus pseudintermedius* canine clinical isolates submitted to a veterinary diagnostic laboratory in South Africa

**DOI:** 10.1371/journal.pone.0290645

**Published:** 2023-08-30

**Authors:** Lufuno Phophi, Mohamed Abouelkhair, Rebekah Jones, Maryke Henton, Daniel N. Qekwana, Stephen A. Kania

**Affiliations:** 1 Department of Biomedical and Diagnostic Sciences, University of Tennessee, College of Veterinary Medicine, Knoxville, TN, United States of America; 2 Vetdiagnostix Veterinary Pathology Services, Midrand, Gauteng, South Africa; 3 Faculty of Veterinary Science, Veterinary Public Health and Epidemiology, University of Pretoria, Pretoria, South Africa; University of Calgary, CANADA

## Abstract

*Staphylococcus pseudintermedius* is an important cause of clinical infections in small-animal-veterinary medicine. Evolutionary changes of strains using multilocus sequence typing (MLST) have been observed among *S*. *pseudintermedius* in European countries and the United States. However, there are limited or no studies on the detection of methicillin resistant *Staphylococcus pseudintermedius* (MRSP) and predominating MLST strains in South Africa. Therefore, this study aimed to determine the molecular epidemiology of *S*. *pseudintermedius* in South Africa. Twenty-six, non-duplicate, clinical isolates from dogs were obtained as convenience samples from four provinces in South Africa. The Kirby Bauer disk diffusion test was used to determine antimicrobial susceptibility. We used Resfinder and the Comprehensive Antibiotic Resistance Database (CARD) to detect antimicrobial resistance genes. Virulence genes were identified using the virulence factor database and Basic Local Alignment Search Tool (BLASTN) on Geneious prime. geoBURST analysis was used to study relationships between MLST. Finally, the maximum likelihood phylogeny was determined using Randomized Axelerated Maximum Likelihood (RAxML). Twenty-three isolates were confirmed as *S*. *pseudintermedius* of which 14 were MRSP. In addition to β-lactam antimicrobials, MRSP isolates were resistant to tetracycline (85.7%), doxycycline (92.8%), kanamycin (92.8%), and gentamicin (85.7%). The isolates harbored antimicrobial resistance genes (*tetM*, *ermB*, *drfG*, *cat*, *aac(6’)-Ie-aph(2”)-Ia*, *ant(6)-Ia*, and *aph(3’)-III*) and virulence genes (*AdsA*, *geh*, *icaA*, and *lip)*. MLST analysis showed that ST2228, ST2229, ST2230, ST2231, ST2232, ST2318, ST2326 and ST2327 are unique sequence types in South Africa. Whereas, previously reported major STs including ST45, ST71, ST181, ST551 and ST496 were also detected. The geoBURST and phylogenetic analysis suggests that the isolates in South Africa are likely genetically related to isolates identified in other countries. Highly resistant MRSP strains (ST496, ST71, and ST45) were reported that could present challenges in the treatment of canine infections in South Africa. Hence, we have gained a better understanding of the epidemiology of MRSP in the African continent, the genes involved in resistance and virulence factors associated with these organisms.

## Introduction

In dogs, *Staphylococcus pseudintermedius* is a commensal bacterium that affects the skin and mucosa and can transiently colonize human beings [1–4]. There is an increasing prevalence of MRSP globally and they have become a threat to the successful treatment of infections in small-animal-veterinary-medicine [1,5–7]. Methicillin resistance in *S*. *pseudintermedius* is attributed primarily to the *mecA* gene harbored within a mobile genetic element called SCC*mec* which encodes penicillin binding protein 2a (PBP2a) [8–10]. The PBP2a allows for cell wall biosynthesis in the presence of most β-lactam antibiotics, thereby inducing resistance [11,12]. *S*. *pseudintermedius* is the most common staphylococcal species isolated from dogs in South Africa [13]. Recently, a study conducted by Prior *et al*. revealed a high occurrence (83.89%) of *S*. *pseudintermedius* and 85.9% prevalence of *mecA* positive carriage among clinical *S*. *pseudintermedius* isolates from five geographical dispersed laboratories in South Africa [14] However, the molecular epidemiology of *S*. *pseudintermedius* isolated from dogs has not been investigated in this country.

Multi-locus sequence typing is a well-established method used to identify dominant MRSP lineages and geographical dissemination of *S*. *pseudintermedius* worldwide. As a result, CC71/ CC258 in Europe, CC68 in the United States, and CC45/CC112 in Asia have been established as dominant clonal complexes in the past [5]. In 2012, Jung-Ho Youn *et al*. reported ST39, ST200, ST54, ST204, ST18 among *S*. *pseudintermedius* isolates from a veterinary hospital in Zambia [15]. In Botswana, ST885 to ST890 were obtained from nasal swab samples collected from healthy dogs [16]. In South Africa, MLST studies of *S*. *pseudintermedius* isolated from dogs are lacking.

There are significant regional differences in patterns of antimicrobial resistance and virulence factors associated with distinct clonal lineages. For example, a low percentage of tetracycline and chloramphenicol resistance was reported from isolates belonging to the CC71 lineage in North America compared to isolates belonging to the CC71 lineage in Europe [1,14]. In France, ST258 MRSP isolates show higher susceptibility to gentamicin, sulfonamides and fluoroquinolones compared to isolates belonging to ST71 [14]. In Australia, ST496 isolates harbor SCC*mec* type Vt whereas ST71 in Europe is associated with SCC*mec* type III [5,17]. Additionally, ST749 in canine Australian isolates carries the *nanB* gene for sialidase, while ST496 in France carries the genes for surface adhesion virulent *spsI* and *spsF* [14,17]. In South Africa, Qekwana *et al*. reported multidrug resistance and increasing levels of resistance to fluoroquinolones and sulfonamides among *S*. *pseudintermedius* isolated from dogs at a veterinary teaching hospital [18]. In Zambia, Jung-Ho Youn *et al*. reported high penicillin and tetracycline resistance among *S*. *pseudintermedius* isolates implicating *tetM* and *blaZ* genes [15]. However, antimicrobial resistance and virulence genes of MRSP clonal lineages isolated from dogs in South Africa have not been demonstrated.

Recent studies in various countries show a changing population structure among *S*. *pseudintermedius* as a result of the acquisition of SCC*mec* and the international movement of animals [5,14,19]. A dearth of information prevents us from understanding how the population structure of *S*. *pseudintermedius* has changed over the years in South Africa. Therefore, the aims of this study were to determine the phenotypic resistance profile, to identify resistant genes, and virulence genes, to determine MLST profiles as well as geoBURST analysis of MRSP clones circulating in South Africa.

## Materials and methods

### Bacterial isolates and species verification

A total of 26, non-duplicate, clinical samples collected in 2021 from dogs were received as convenience samples from a veterinary diagnostic laboratory in South Africa, Gauteng. The samples were obtained from Johannesburg, Middleburg, Cape Town, Port Elizabeth, Pietermaritzburg, and Randburg and submitted for diagnostics analysis at a veterinary diagnostic laboratory. Clinical isolates were taken from post-operative infections, cystitis, otitis, wound, abdominal fluids, left front limb, pyoderma, and hock hygroma. Isolates were then shipped to the University of Tennessee Veterinary College, bacteriology lab in transport media for further analysis. *S*. *pseudintermedius* isolates were inoculated onto Columbia blood agar plates containing 5% sheep blood (Remel) and incubated overnight at 37°C in 5% CO_2_. The isolates were identified as *S*. *pseudintermedius* using the matrix-assisted laser desorption/ionization-time of flight mass spectrometry (MALDI-TOF MS, Bruker). *S*. *pseudintermedius* species assignment was made when the log(score) values were ≥ 2 [20].

### Antimicrobial susceptibility

Antimicrobial susceptibility tests were performed on all confirmed *S*. *pseudintermedius* isolates. The Kirby-Bauer disk diffusion method was used and interpreted as recommended by the most current Clinical and Laboratory Standards Institute (CLSI) veterinary guidelines available at the time of the study [20].Mueller Hinton agar plates and antimicrobial disks were obtained commercially (BD Diagnostic Systems, Sparks, MD and Remel) The antimicrobials tested were penicillin (P) 10μg, clindamycin (DA) 2μg, kanamycin (K) 30μg, erythromycin (E) 15μg, gentamicin (CN) 10μg, oxacillin (OX) 1μg, tetracycline (TE) 30μg, minocycline (MH) 30μg, doxycycline (DO) 30μg, chloramphenicol (C) 30μg, cephalothin (KF) 30μg, vancomycin (VA) 30μg, streptomycin (S) 10μg, rifampin (RD) 5μg, marbofloxacin (MAR) 5μg, amoxicillin-clavulanic acid (AMC) 20μg, fosfomycin (FOS) 20μg, linezolid (LZD) 30μg, cefpodoxime (CPD) 10μg, cefoxitin (FOX) 30μg, and sulfamethoxazole-trimethoprim (SXT) 1.25/23.75ug. Methicillin resistance was determine by oxacillin resistance based on interpretation of the Clinical Laboratory Standard Institute for veterinary medicine (CLSI Vet, 2018) [20]. Interpretation of vancomycin was based on previously described criteria for *S*. *aureus* by Rezaeifar *et al*. [21]. Additionally, Fosfomycin was interpreted using recommendation from Comité de l’Antibiogramme de la Société Française de Microbiologie (CASFM, 2013) [22]. The CLSI M100 was used for the interpretation of linezolid, streptomycin, and kanamycin [23]. Whereas cephalothin, minocycline and amoxicillin-clavulanic acid was interpreted according to the CLSIVET01S2 [24]. Erythromycin and clindamycin disks were placed approximately 15 mm apart so interpretation of inducible clindamycin resistance could be made for isolates that are resistant to erythromycin but otherwise susceptible to clindamycin. Isolates in the intermediate category were classified as resistant for the purpose of analysis. Isolates that were resistant to three or more antimicrobial drug classes were defined as “multidrug-resistant”.

### DNA extraction and whole genome sequencing

Three or four bacterial colonies were suspended in 3 ml of sterile tryptic soy broth, incubated at 37 C° overnight, and a commercial kit (UltraClean→ Microbial DNA isolation Kit, Qiagen) was used for DNA extraction. The concentration was measured using a nanodrop and Qubit 4 fluorometer (Fisher, USA). Sequencing libraries were constructed using a Nextera DNA sample prep kit (Illumina, Inc., USA) according to the manufacturer’s instructions. The genomes were sequenced using a MiSeq platform (Illumina, Inc.) with a single end read length of 150 bp at the University of Tennessee Immunology Laboratory. Whole genome sequences were assembled using SPAdes (http://bioinf.spbau.ru/spades). Annotation and analysis of the genomes was performed using the PATRIC software [25]. The genomes were then submitted to the NCBI Genbank as Bio project https://www.ncbi.nlm.nih.gov/bioproject/PRJNA892647.

### Molecular epidemiology

ResFinder (genomic epidemiology.org), CARD (Comprehensive Antimicrobial Resistance Database, https://card.mcmaster.ca/) databases on PATRIC were used to identify resistance genes. Virulence genes were detected using the Virulence Factor Database (VFDB), additionally the Geneious prime was used for BLASTN analysis of virulence genes [26].

### Population structure analysis

Multilocus sequence typing (MLST) of seven housekeeping genes (*tuf*, *cpn60*, *pta*, *purA*, *fdh*, *ack*, and *sar*) was used to determine the sequence type (ST) of each isolate as we described previously [27]. The sequence types were assigned by comparison with the allele sequences available in the PubMLST database. Isolates with new combinations of alleles were submitted to the MLST database curator Vincent Perreten for assignment. Using entries from the global PubMLST *S*. *pseudintermedius* database, we ascertained the clonal relationships of the sequence types obtained in this study through goeBURST clustering analysis on PHYLOViZ [28]. To further investigate the STs relationships compared to the major STs identified in the United States, Europe and Asia (ST258, ST45, ST71, ST68), Botswana (ST888, ST885, ST887, ST886, ST889) and Kenya (ST590, ST591, ST592, ST593, ST594) together with STs identified in South Africa.

## Phylogeny

The Global protein families (PGFams) in PATRIC were used to identify 655 protein families from genes that were present as a single copy per genome [25]. For each of the chosen genes, both the gene (nucleotide) and encoded protein (amino acid) sequences were analyzed. MUSCLE was used to align protein sequences and nucleotide sequences [29]. The sequences were concatenated into one alignment using a phylip formatted file. The maximum likelihood phylogeny was then generated using Randomized Accelerated Maximum Likelihood (RAxML) with a general time-reversible nucleotide substitution model and four gamma categories for rate heterogeneity [30]. The resulting newick file was viewed in FigTree v1.4.4. Previously reported genomes reported worldwide were obtained from GenBank and used for phylogenetic analysis with isolates reported from South Africa (Table 1).

**Table 1:** Genomes used for phylogenetic analysis with genomes reported from South Africa in this study.

Strain	GenBank Accession	Genome Length(bp)	Contigs	Country	ST
KCTC 43134	CP045084	2611104	1	South Korea	585
HKU10-03	CP002439	2617381	1	Hong Kong	308
E104	LAWU00000000	2757000	49	Germany	N/A
SP79	AP019372	2509706	1	Japan	2141
ED99	CP002478	2572216	1	Germany	25
LMG 22219	MLGE00000000	2523112	50	United States	N/A
NA45	CP016072	2841212	1	United States	84
ST496-1	QQPB00000000	2745362	467	Australia	496
ST64-4	QEJG00000000	2681612	463	Australia	64
E140	ANOI00000000	2769458	1	Denmark	71
2080722072011	PEOJ00000000	2571729	101	Netherlands	45
2121224012011	PEPS00000000	2650237	98	Netherlands	258
SL/114	MQND00000000	2590176	51	Sri Lanka	45
ST258 1	QEJD00000000	2620567	1441	Australia	258
VTH551	RJQE00000000	2637925	32	United States	258

### Statistical analysis

All proportions and 95% confidence intervals in this study were performed using SAS ® 9.4 (SAS Institute Inc., Cary, NC, USA).

## Results

### Isolate collection

Based on MALDI-TOF, twenty-three isolates were confirmed as *S*. *pseudintermedius* and distributed geographically as shown in Table 2. The samples represent 4 provinces in South Africa (Gauteng, Kwazulu-Natal, Western Cape, and Eastern Cape) that were submitted for diagnostics at a Vet diagnostic laboratory. Nine of the isolates were methicillin susceptible (MSSP) and 14 of the isolates were MRSP. Seventeen different STs were identified in the isolates from South Africa. Eight *S*. *pseudintermedius* alleles were unique and were assigned STs in this study (ST2232, ST2229, ST2228, ST2231, ST2230, ST2318, ST2326, ST2327). Five of the MRSP isolates belonged to ST496, ST1308, ST45, ST181 and ST1111. Whereas seven of the MSSP isolates were previously reported on the pubMLST database (ST301, ST551, ST261, ST121, ST496, ST71, ST181 and ST1431).

**Table 2:** Characteristics of 23 *S*. *pseudintermedius* isolated from canine clinical samples submitted to a veterinary diagnostic laboratory in South Africa, 2021.

Isolate	Age of Dog (Year)	Collection Site	Location of Collection (City)	Methicillin Resistance	ST	Accession No
20VMG1528	5y	Post-op	Johannesburg	S	301	SAMN31393558
20VMG1536	6y	Skin	Stella	S	551	SAMN31393559
20VMG1580	7y	Ear	Randburg	S	261	SAMN31393560
21VMG0143	2y	Skin	Randburg	R	496	SAMN31393561
21VMG0271	7y	Skin	Randburg	R	1308	SAMN31393562
21VMG0402	6y	Hock-hygroma	Johannesburg	R	2232	SAMN31393563
21VMG0561	8y	Skin	Port Elizabeth	S	2230	SAMN31393564
21VMG0581	5y	Wound	Johannesburg	R	2229	SAMN31393565
21-VMG0567	5y	Post-op	Johannesburg	S	121	SAMN31393566
21-VMG0568	8y	Skin ulcers	Johannesburg	S	496	SAMN31393567
21VMK0081	3y	Mastitis	Pietermaritzburg	S	71	SAMN31393568
21VMK0268	8y	Toe	Umvoti	R	261	SAMN31393569
21VMK0323	4 months	Pyoderma	Durban	R	2228	SAMN31393570
0445	N/A	N/A	N/A	R	2326	SAMN31393571
21VMK0532	8y	Pyoderma	Salt Rock	R	45	SAMN31393572
21VMG0811	2y	Post-op	Johannesburg	R	2231	SAMN31393573
0536	N/A	N/A	N/A	S	181	SAMN31393574
21–2683	N/A	Lip fold	Cape Town	S	1431	SAMN31393575
21–3244	5y	Wound	Cape Town	R	496	SAMN31393576
21–3512	3y	Pyoderma	Cape Town	R	1111	SAMN31393577
21–5383	2y	Post-op	Cape Town	R	496	SAMN31393578
21–3929	2y	Cystitis	Cape Town	R	181	SAMN31393579
21–5495	2y	Nail Bed infection	Cape Town	R	2228	SAMN31393580

MRSP, methicillin resistant *S*. *pseudintermedius*, MSSP, methicillin susceptible *S*. *pseudintermedius*, ST, Sequence type.

### Antibiotic resistance

All isolates were resistant to at least one antibiotic whereas 87% of the isolates were multidrug resistant (MDR) (i.e., resistance to 3 or more classes of antimicrobials) (Table 3). Most of the isolates were resistant to penicillin (95.6%), streptomycin (91.3%), kanamycin (78.2%), trimethoprim/sulfamethoxazole (73.9%), and cefpodoxime (60.8%). The level of tetracycline and doxycycline resistance among the isolates was similar (69.5%) whereas minocycline resistance was 43.3%. Low resistance was observed to cephalothin (17.4%), chloramphenicol (17.4%), fosfomycin (17.4%) and cefoxitin (17.4%). None of the isolates were resistant to vancomycin, linezolid, or rifampin. All MRSP isolates were resistant to penicillin and cefpodoxime. MRSP isolates exhibited high proportions of resistance to doxycycline (92.8%), kanamycin (92.8%), tetracycline (85.7%), streptomycin (85.7%) and trimethoprim/sulfamethoxazole (85.7%) compared to low proportion of MSSP resistance to doxycycline (33.3%), kanamycin (55.6%), tetracycline (44.4%) and trimethoprim/sulfamethoxazole (55.6%).

**Table 3:** Antimicrobial resistance patterns of *S*. *pseudintermedius* isolated from canine clinical samples submitted to a veterinary diagnostic laboratory in South Africa, 2021.

		*S*. *pseudintermedius*(n = 23)			MRSP(n = 14)			MSSP (n = 9)		
		Percent (%)	95%CI^a^		Percent (%)	95% CI^a^		Percent (%)	95% CI^a^	
Group	Antibiotic		Lower	Upper		Lower	upper		Lower	Upper
Lincosamide	Clindamycin	39.1	40.9	80.8	57.1	16.9	68.7	11.11	68.4	100
Penicillins	Penicillin	95.6	87.1	100	100	100	100	88.89	68.4	100
	Amoxicillin-clavulanate acid	21.7	61.4	95.2	35.7	39.2	89.3	0	100	100
	oxacillin	60.8	19.2	59.1	100	100	100	0	100	100
Cephalosporin	cefoxitin	17.4	67.1	98.1	28.5	47.7	95.1	0	100	100
	Cefpodoxime	60.8	19.1	59.1	100	100	100	0	100	100
	Cephalothin	17.4	67.1	98.1	28.5	47.7	95.1	0	100	100
Tetracyclines	Tetracycline	69.5	11.6	49.2	85.7	67.3	100	44.4	23.1	88
	Minocycline	43.4	36.2	76.7	57.1	83.1	31.2	22.2	50.6	100
	Doxycycline	69.5	11.6	49.2	92.8	79.3	100	33.3	35.8	97.5
Phenicols	Chloramphenicol	17.4	67.1	98.1	21.4	57.1	100	11.1	68.4	100
Glycopeptide	Vancomycin	0	100	100	0	100	100	0	100	100
Aminoglycoside	Streptomycin	91.3	79.7	100	85.7	67.3	100	100	100	100
	Gentamicin	56.5	23.2	63.7	85.7	67.3	100	11.1	68.4	100
	Kanamycin	78.2	61.4	95.1	92.8	79.3	100	55.6	23.1	88
Macrolide	Erythromycin	39.1	40.9	80.8	57.1	16.9	68.7	11.1	68.4	100
Fluoroquinolone	Marbofloxacin	34.7	45.7	84.6	57.1	16.9	68.7	0	100	100
Rifamycin	Rifampin	0	100	100	0	100	100	0	100	100
Phosphonic	Fosfomycin	17.4	67.1	98.1	28.5	47.7	95.1	0	100	100
Oxazolidinones	Linezolid	0	100	100	0	100	100	0	100	100
Folate inhibitor	Trimethoprim/sulfamethoxaole	73.9	8.14	44	85.7	67.3	100	55.6	11.9	76.9

^a^95% CI = 95% confidence interval.

### Antibiotic resistant genes

The *blaZ* gene which encodes for narrow spectrum beta-lactamases was detected in all but one isolate (Table 4). Sixteen of the *S*. *pseudintermedius* isolates harbored *tetM* resistance genes whereas one isolate harbored *tetK*. The *dfrG* gene conferring the trimethoprim-sulfamethoxazole resistance phenotype was detected in 14 isolates. Most (11/23) of the isolates in this study harbored macrolide resistance, *ermB* genes. Chloramphenicol acetyltransferase genes (*catpC221*) were detected in six isolates. Isolates harbored *aac6-aph2* (14/23), *ant6-Ia* (9/23) and *aph3-III* (9/23) that confer resistance to acetyltransferase, nucleotidyl transferase and phosphotransferase, respectively.

**Table 4:** Antimicrobial resistance genes of *S*. *pseudintermedius* isolated from canine clinical samples submitted to a veterinary diagnostic laboratory in South Africa, 2021.

Isolate	*aac(6’)-Ie-aph(2”)-Ia*	*ant(6)-Ia*	*aph(3’)-III*	*blaZ*	*catpC221*	*dfrG*	*ermB*	*mecA*	*tetK*	*tetM*
20VMG1528										
20VMG1536										
20VMG1580										
21VMG0143										
21VMG0271										
21VMG0402										
21VMG0561										
21VMG0581										
21VMG0567										
21VMG0568										
21VMK0081										
21VMK0268										
21VMK0323										
0445										
21VMK0532										
21VMG0811										
0536										
21–2683										
21–3244										
21–3512										
21–5383										
21–3929										
21–5495										

### Virulence genes

**Fig 1:**
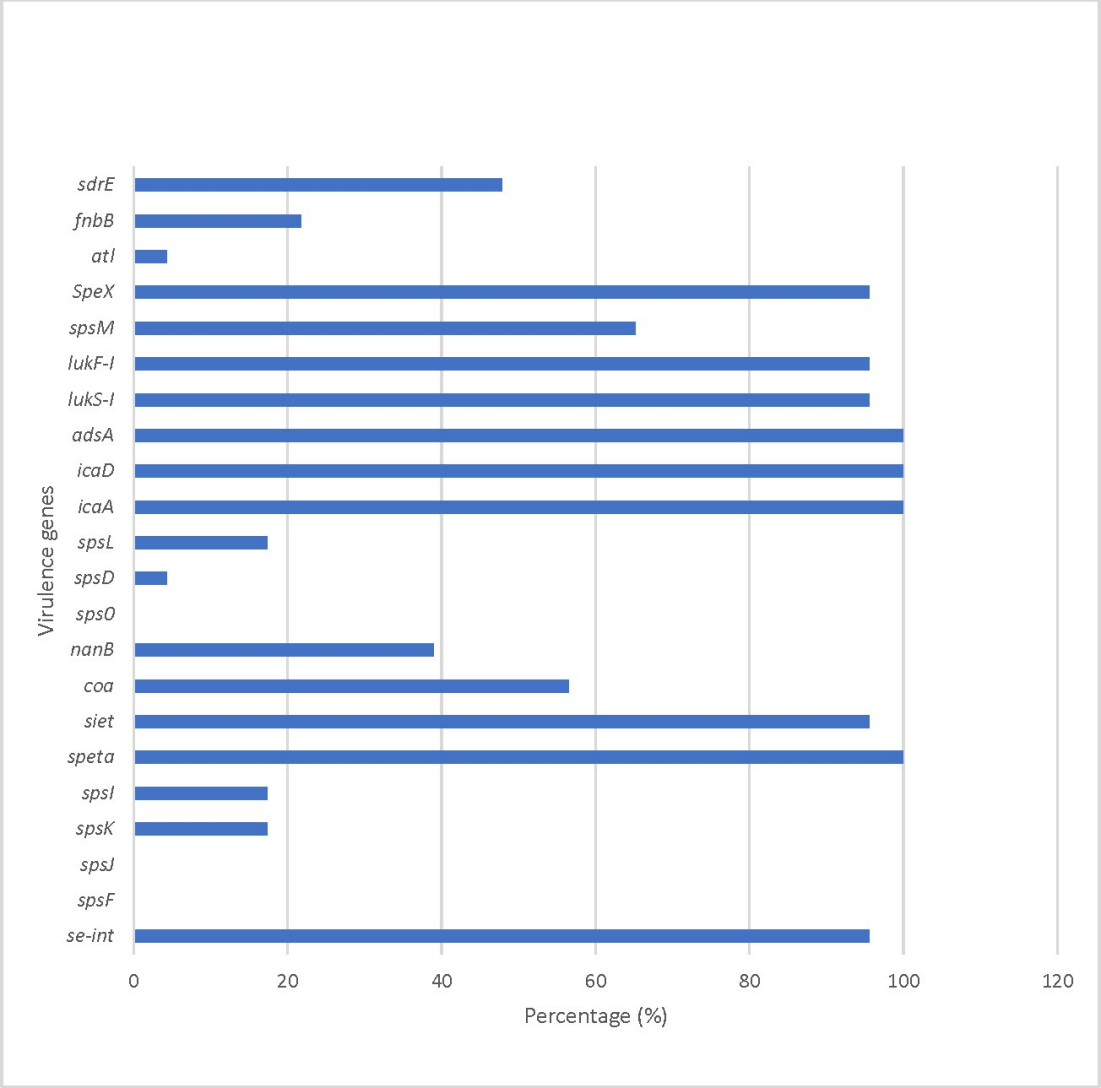
Virulence genes detected among *S*. *pseudintermedius* isolates submitted to a veterinary diagnostic laboratory in South Africa, 2021. *S*. *pseudintermedius* cell wall anchored proteins genes (*spsM*, *spsL*, *spsD*, *spsO*, *spsI*, *spsK*, *spsF*), fibrinogen binding protein gene (*fnbB*), Intercellular adhesion (*icaA*, *icaB*), leukotoxin (*lukF-I*, *lukS-I*), ser-asp rich fibrinogen binding protein (*sdrE*), immune evasion (*adsA*), exfoliative toxin (*se-int*, *SpeX*, *speta*, *siet*) and exoenzyme (*coagulase*, *nanB*).

All the isolates were positive for immune evasion (*adsA*), exfoliative toxin (*speta*) and intracellular adhesion gene (*icaA and icaD*) (Fig 1). Whereas none of the isolates harbored the *spsO*, *spsJ* and *spsF* virulence genes. The *seh* and *seg* canine enterotoxin gene was only identified in MRSP isolates while the *atl* and *spsD* were identified among MSSP isolates. Isolates belonging to ST2228 and ST181 harbored the *spsI* gene compared to ST496. The ST71 isolate in this study did not harbor the *lukS-I* and *lukF-I* virulent gene compared to other isolates in the study. Compared to ST551, ST496 harbored *sdrE*, *nanB* and *spsM* virulent genes.

### GeoBURST analysis

**Fig 2:**
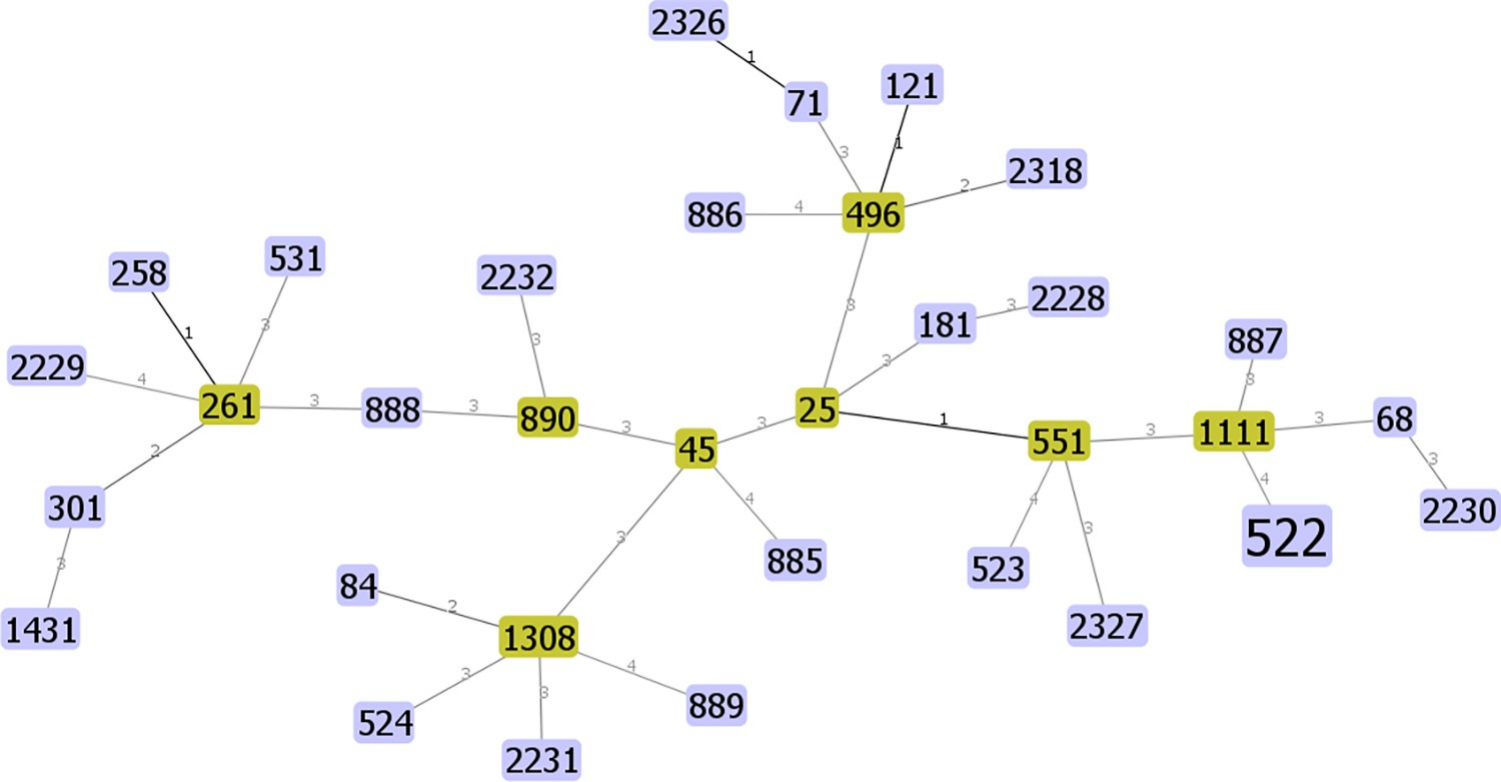
Clonal relationship of MLST using isolates identified in South Africa and major STs identified worldwide. The geoBURST full MST algorithm showed links connected by SLV, DLV, TLV and 4 link variants. Each node represents a ST. The size of the node denotes the sample size of each ST. The distance between each node is represented by 1 = SLV, 2 = DLV, 3 = TLV and 4 = four locus variants. The predicted founder clonal complexes are represented as green.

The isolates representing the assigned STs (ST2232, ST2231, ST2228, ST2229, ST2230) were singletons and were not related to the STs in the database (Fig 2). These STs were part of a branch located very far from the other isolates indicating a distant evolutionary relationship. To further investigate clonal relationships, the geoBURST full MST algorithm was used for STs identified in Kenya, Botswana, and South Africa with major STs identified in the United States (ST68), Europe (ST71 and ST258) and Asia (ST45). Four links were observed at level 1, 3 links at level 2, 19 links at level 3 and 6 links at level 4. None of the links connected STs in this study with those identified in Botswana and Kenya. No clonal complex could be identified from these STs because none of the CCs contained more than 3 STs with single locus variant (SLV = 1). However, ST2326 assigned in this study had a single locus variant to ST71 while ST2318 had a double locus variant to ST496. None of the isolates assigned in this study were closely related to ST68, ST45 and ST258 with a single locus variant.

### Phylogeny

**Fig 3:**
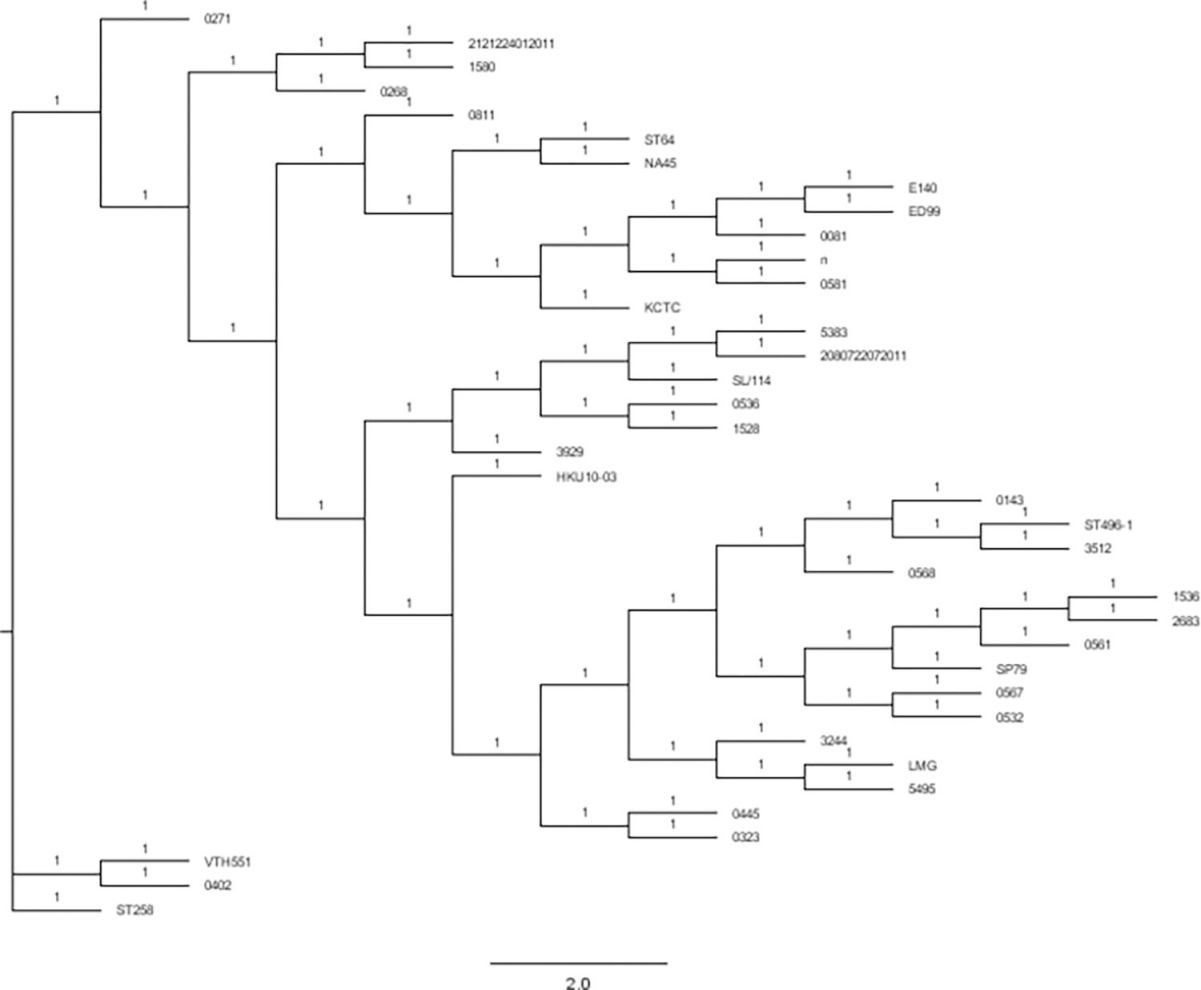
Phylogenetic tree based on the time-reversible nucleotide substitution model and four gamma categories for rate heterogeneity of *S*. *pseudintermedius* isolates. The phylogenetic tree includes isolates of the present study and selection of previously sequenced genomes representing phylogenetic diversity found across the species.

A phylogenetic tree that included 15 previously sequenced *S*. *pseudintermedius* genomes and the 23 isolates in this study were generated (Fig 3). The phylogenetic analysis showed that clustering of some isolates identified in this study were not monophyletic, with 21VMG0402 representing ST2232 assigned in this study separated from the other isolates.

## Discussion

Methicillin resistant *S*. *pseudintermedius* is the predominant cause of clinical pyoderma in dogs [10,19,27]. The outcome of infection is dependent on the antimicrobial resistance profile and virulence factors associated with different sequence type [10,31,32]. In this study we investigated the molecular epidemiology of *S*. *pseudintermedius* isolated from dog samples submitted to a veterinary diagnostic laboratory in South Africa.

All isolates in this study were resistant to at least one antimicrobial, 87% were MDR, whereas 60.8% were MRSP. The prevalence detected in this study was relatively low when compared to high levels (85.9%) observed among dogs with pyoderma and otitis in South Africa [33]. High levels of MRSP were detected in this study compared to 14% reported among clinical isolates of dogs in Finland and 30.8% reported among clinical isolates in the United States [32,34]. In Finland, MRSP was determined among 1958 clinical *S*. *pseudintermedius* isolates collected from private clinics and veterinary teaching hospitals [32]. Whereas in the USA, Lord *et al*. reported MRSP among canine clinical specimens processed at the University of Tennessee College of Veterinary Medicine between January 1, 2006 and December 31, 2017 [34]. Therefore, due to the low number of samples collected in this study, the proportion of MRSP and MSSP are not a representation of the distribution of *S*. *pseudintermedius* in the South African dog population. However, the high proportion of MRSP detected in this study suggests that the detection of MRSP in South Africa is concomitant with global MRSP trends.

Resistance to antimicrobials was higher among MRSP compared to MSSP isolates. In addition to β-lactam resistance, MRSP isolates in this study were resistant to tetracyclines, aminoglycosides, folate inhibitors, macrolide and lincosamides. This is similar to results reported among MRSP isolates in Argentina and Finland [7,35]. In a previous study, Qekwana *et al*. reported a significant increase in the proportions of *S*. *pseudintermedius* resistant isolates to trimethoprim-sulfamethoxazole, clindamycin and orbifloxacin between 2007 and 2012 in South Africa (13). Canine antibiotic resistance in South Africa may be influenced by changes in veterinary prescription practices. Therefore, more studies elucidating the implications of antibiotic use and MRSP in South Africa should be a priority for future studies.

Similar to other studies, the genes responsible for penicillin, tetracyclines, erythromycin and trimethoprim-sulfamethoxazole (*blaZ*, *tetM*, *ermB*, and *dfrG*) were found to be the predominant genes encoding resistance in *S*. *pseudintermedius*. Resistance to aminoglycosides was associated with the presence of adenyl nucleotidyl transferase gene (*ant(6)-Ia*), phosphotransferase gene (*aph(3’)-III*), and the acetyltransferase/phosphotransferase gene (*aac(6’)-Ie-aph(2”)-Ia*) in all isolates except five that only harbored the *aac(6’)-Ie-aph(2”)-Ia* gene. Other studies have reported a strong correlation between the resistant phenotypes and resistance genes detected among *S*. *pseudintermedius* isolates [7,14,36]. It has been suggested that inconsistencies between resistant genes present among susceptible isolates could be attributed to insertion, deletion or mutation in the genes involved [37]. Furthermore, future studies should analyze the promoter sequences of resistance genes from isolates showing susceptibility despite an intact resistance gene.

The prevalence of virulence genes among *S*. *pseudintermedius* isolates in this study are similar to those reported in other studies [14,38,39]. No lineage associated virulence genes were identified in this study. However, compared to ST551, ST496 isolates harbored *sdrE* (cell wall adhesins), *nanB* (putative sialidase toxin) and *spsM* (cell wall anchor protein). The *nanB* gene contributes to colonization by offering a carbon source for growth, forming biofilms, or increasing adherence if receptors on the host are exposed [40]. The *spsM* cell wall protein and *sdrE* have been associated with mediating binding, invasion, and degradation of epithelial cells [40,41]. Whereas the isolate belonging to ST71 in this study did not harbor the leukocyte genes (*LukS-I* and *LukF-I*) that are responsible for destruction of canine polymorphonuclear leukocytes (PMNs) by pore formation and cell lysis [42]. The success of *S*. *pseudintermedius* lineages has previously been associated with variation in the surface proteins. Therefore, more studies analyzing the functional expression of virulence genes among canine *S*. *pseudintermedius* isolates could inform on the successful lineages in South Africa.

geoBURST analysis could not identify a main clonal founder and clonal structure of MSSP and MRSP isolates in South Africa. In previous studies, MRSP isolates were associated with fewer clones than MSSP isolates, which had relatively less clonal expansion [1,5,19,36]. This finding could suggest that the strains share a very distant relationship and do not share any recent common ancestor. The diversity observed in this study suggests that the *S*. *pseudintermedius* population structure isolated from dogs in South Africa could be non-clonal. In comparison, Dos Santos *et al*. concluded that the population of *S*. *pseudintermedius* was weakly clonal due to the presence of recombination among STs worldwide [5]. The PubMLST website had not previously reported ST2232, ST2231, ST2228, ST2229 and ST2230. These clones represent locally evolved clones since they were located on a very distant branch compared to STs in the database. However, ST496, ST71, ST551, ST45 and ST181 detected in this study have been reported worldwide [5,14,19,43]. These clones could have been transported from the countries they were first identified in. ST496 was identified as a novel clonal lineage in Australia in 2018 whereas ST551 was first identified in Poland in 2016 [19,44]. Of concern, ST496 has shown significant antibiotic resistance to veterinary antibiotics and contains virulence genes such as *spsI* and *spsF* associated with adhesion to the extracellular matrix [14]. The isolate in this study representing ST551 harbored *tetK* which encodes tetracycline resistance through efflux pumps. This is a unique feature that has been identified for ST551 among MRSP isolated from dogs in Poland and Slovenia [43,44]. Hence, determining clinical implications of dominant *S*. *pseudintermedius* strains in South Africa could help in successful treatment of canine infections.

## Conclusions

This is the first report addressing the phenotypic and genotypic characterization of canine *S*. *pseudintermedius* isolates submitted to a veterinary diagnostic laboratory in South Africa. The results from this study suggest the presence of a highly diverse clonal population structure of *S*. *pseudintermedius*. Additionally, highly resistant clonal lineages such as ST71, ST45, ST551 and ST496 were detected that pose a threat to the successful treatment of MRSP infections in South Africa. The collection of *S*. *pseudintermedius* and MRSP in South Africa is underrepresented compared to worldwide continental reports. However, information on antimicrobial resistance and molecular epidemiology of MRSP in South Africa is vital to obtain a universal understanding of this organism. Therefore, future studies should investigate the occurrence and patterns of multidrug resistant MRSP isolated from canine clinical samples representing a larger population of dogs in South Africa.

## Supporting information

S1 Table Virulence genes identified in each South African isolate. (XLSX)

S2 Table Alleles for each South African isolate used to determine MLST profiles and for geoBURST analysis. The alleles were assigned using https://pubmlst.org/organisms/staphylococcus-pseudintermedius.
(TXT)
